# Virtual Examination Techniques, Clinical Effectiveness, and Future Directions of Telemedicine in Orthopedic Practice: A Narrative Review

**DOI:** 10.7759/cureus.97548

**Published:** 2025-11-23

**Authors:** Ahmed Swealem, Mohamed Wahb, Mohamed Abosheisha, Amr Basha, Fahad Al-Hasani, Ahmed Moustafa Mahmoud Shaheen, Mustafa Alqasem, Mohamed Fahmy, Mohamed Dief Allah, Fadi Nabil Rofaeel Ibrahim

**Affiliations:** 1 Orthopedics, Southmead Hospital, North Bristol NHS Trust, Bristol, GBR; 2 Trauma and Orthopaedics, Wexham Park Hospital, Frimley Health NHS Foundation Trust, Slough, GBR; 3 General Surgery, Wirral University Teaching Hospital NHS Foundation Trust, Wirral, GBR; 4 General Surgery, Warwick Medical School, University of Warwick, Coventry, GBR; 5 Trauma and Orthopaedics, Watford General Hospital, West Hertfordshire Teaching Hospitals NHS Trust, Watford, GBR; 6 Trauma and Orthopaedics, Royal Glamorgan Hospital, Ynysmaerdy, GBR; 7 Plastic Surgery, North Bristol NHS Trust, Bristol, GBR; 8 General Surgery, Northumbria Healthcare NHS Foundation Trust, North Tyneside, GBR; 9 Urology, Glan Clwyd Hospital - Betsi Cadwaladr University Health Board, Bodelwyddan, GBR; 10 Cardiology, El Moqattam Hospital for Medical Insurance, Cairo, EGY; 11 Plastic and Reconstructive Surgery, North Bristol NHS Trust, Bristol, GBR

**Keywords:** artificial intelligence in telemedicine, orthosurgery, teleconsultation, telerehabilitation, video telemedicine

## Abstract

Telemedicine adoption has made notable changes in orthopedic practice. It was greatly expanded with the COVID-19 pandemic, with the adoption across subspecialties offering comparable diagnostic accuracy, patient satisfaction, and safety to traditional visits. This narrative review summarizes the current applications of telemedicine and future directions in orthopedic telemedicine. Virtual examinations for the elbow, shoulder, hip, and knee have been successfully modified for remote assessment. The modifications of some tests now allow patients to perform self-tests under physician guidance using household tools. Randomized controlled trials (RCTs) demonstrate that telehealth consultations are not inferior to in-person evaluations in clinical decision-making. Limitations include the inability to replicate certain hands-on maneuvers, disparities in digital access among minority and low-income populations, and variable patient adherence. To optimize remote care, standardized telehealth protocols, improved image transmission, and targeted patient preparation are required. Artificial intelligence (AI) and large language models (LLMs) promise to enhance virtual care. Telemedicine is positioned to become a permanent, high-quality complement to in-person orthopedic practice, expanding global access to efficient and patient-centered musculoskeletal healthcare.

## Introduction and background

Telemedicine is now a promising service in musculoskeletal care, as it offers virtual physical examinations, enhances patient education, and provides better and wider availability of treatment and telerehabilitation [1]. Telemedicine provides significant benefits for musculoskeletal care, enabling remote consultation, management, and rehabilitation. Its use surged by 75% in U.S. hospitals between 2020 and 2021 [2]. 

The COVID-19 pandemic led to a reduction in patients' visits to clinics and a simultaneous rise in remote telehealth consultations [3]. The pandemic-related social distancing and in-person visit restrictions encouraged both physicians and patients to use telehealth. Research indicates that telehealth appointments reduce visit and wait times, lower healthcare costs, and achieve patient satisfaction comparable to traditional in-person visits [4-7]. However, orthopedic surgeons did not widely use telemedicine [8].

Technological advancements have given rise to intuitive, web-based videoconferencing systems, which serve to facilitate good and effective interactions between patients and their healthcare providers even across long distances [9]. A randomized controlled trial (RCT) found that 98% of telehealth orthopedic surgeons rated their examination performance and ability as good or very good, showing no significant difference from face-to-face visits and no safety concerns [10]. Additional research indicates that telemedicine can serve as a substitute for traditional in-person appointments for orthopedic outpatients [11-14]. However, many clinicians consider a thorough physical examination essential for clinical evaluation, and a current concern with telehealth is whether it allows for an effective examination between doctors and patients [15-19]. 

## Review

Overview of telemedicine in the orthopedics subspecialty 

Telemedicine is now gaining attraction and widespread adoption, especially after the COVID pandemic. There is an increasingly comprehensive understanding developing regarding the optimal methods for understanding diseases using it. This evolving comprehension also extends to how these virtual interactions significantly affect the overall delivery of patient care [20].

We can note that face-to-face examination and history taking are clearer and straightforward than telemedicine practice, so telehealth practical tests should be adjusted. This challenge is especially pronounced in the various subspecialties of orthopedics, each of which utilizes its own distinct and subtle examination techniques to be suitable for each situation [21]. 

First, patients should write a new patient form, detailing their medical history and current concerns. Clinicians can then review this information to guide the interview. Patients should also receive instructions before the visit on appropriate clothing or required exposure, examination area, and camera/self-positioning to prevent delays [22].

Essential patient details must be gathered, including their primary concern, current illness history, past medical and surgical history, allergies, current medications, social history, and a review of systems. In addition, patients should report any family history of orthopedic or rheumatologic conditions [23].

Elbow Physical Exam With Telemedicine

Men should remove their shirts or wear a tank top, while women should wear a tank top or sports clothes. The patient requires 10-15 feet of clear space to move away from the camera. The examination will involve standing and sitting according to the examiner's questions, with the patient 4-5 feet from the camera, which should be at elbow height. A chair, grocery bags, and eight 16-ounce soup cans are standard household items required for the exam [22]. Several physical examination techniques for the elbow have been successfully adapted for patients to use, facilitating diagnosis in telemedicine settings. For ligamentous instability, the milking maneuver and moving valgus stress tests serve as highly effective initial examination and detection for ulnar collateral ligament pathology [24,25]. 

Patients can perform self-screening for certain orthopedic conditions remotely without help. For example, a modified chair push-up test helps identify posterolateral rotatory instability, and a patient-performed valgus extension overload test is also available. Tendon issues like lateral epicondylitis can be screened using modified Maudsley and chair tests with household items, while the hook test and passive forearm pronation test can be self-administered to check for distal biceps tendon ruptures with appropriate guidance. Nerve compression syndromes can also be detected with self-tests, including the passive pronation with wrist flexion test for radial tunnel syndrome, an adapted resisted supination test for posterior interosseous nerve syndrome, and the patient-performed Tinel sign for pronator syndrome. All of these tests are adjusted and adapted to be performed by patients [22].

Shoulder Physical Exam With Telemedicine

The shoulder examination must incorporate a cervical spine assessment, checking range of motion and pain location, and conducting the Spurling test to comprehensively cover all aspects of the suspected pathology [17,26]. 

Clinicians should look for muscle deformities, skin problems, swelling, or bruising. Patients should turn to allow the camera to view all sides of the shoulders, with the latter view being useful for identifying rotator cuff atrophy. Patients should also point to their maximal pain location. Range-of-motion testing should assess for symmetry and pain-related limitations, with specific views for different movements: forward flexion from the side, external rotation and abduction facing the camera, and internal rotation with the patient's back to the camera. Technologies like smartphone apps, virtual goniometers, accelerometers, and gyroscopes can aid in evaluating shoulder range of motion [27-29].

Patients can independently perform sensory tests and peripheral vascular examinations, comparing the results to their free limb to inform the physician. For sensory testing, patients will report any numbness, burning, or tingling, and may be asked about specific areas. For the peripheral vascular exam, patients can assess if one hand feels colder than the other and check capillary refill. Shoulder injuries are often linked to scapulothoracic kinematics and dynamic motion [18,30-32].

The Kibler test checks the function and movement of both shoulder blades during arm elevation in both the sagittal and scapular planes; it classifies the motion as either "normal" or indicative of "scapular dyskinesis [33].

We can make categories for specialized tests such as impingement, glenohumeral instability, acromioclavicular (AC) joint arthrosis, biceps-labrum complex (BLC) disease, thoracic outlet syndrome, and generalized ligamentous laxity [17-19,34]. Patients can perform these tests on their own after slight education, with only slight modifications to the original techniques [35].

Hip Physical Exam With Telemedicine

Diagnosing hip and groin pain in young adults and adolescents has traditionally been challenging and may require several visits and investigations. On average, patients consulted three healthcare providers before receiving an accurate diagnosis [36,37]. Many hip examination techniques can be adapted for virtual performance. A recent study by Owusu-Akyaw et al. [38] highlighted the diagnostic utility of self-administered hip exams.

To examine the hip, the patient should be at a suitable distance, and it involves inspecting the hip region, overall alignment of the spine and lower extremities, and gait. Patients should turn to allow for full inspection of the skin and musculature. The camera view needs to accommodate several stride lengths for gait analysis and be adjusted to observe knee and foot progression for femoral version assessment. While standing, patients can palpate the area of maximum tenderness that is causing significant complaint, with groin pain [39]. 

The seated part of the exam assesses hip range of motion (ROM) and neurovascular status. The patient sits with hips at 90 degrees and performs five-second rotations, evaluating symmetry and pain at the maximum point and measuring with a virtual goniometer [40].

During the supine position, a full ROM for the hip is assessed, along with the use of provocative tests. Hip flexion is most effectively evaluated in this position, and a hip flexion contracture can be identified through the Thomas test [41]. When the patient is in the supine position, he will raise his leg to create a significant force across the hip joint, potentially several times their body weight, which may lead to hip pain [39].

To examine the peritrochanteric region, patients can be on their free side during the lateral part of the examination. They can be prompted to feel their greater trochanter for potential trochanteric bursitis or tears in the gluteus medius and minimus tendons that will appear as a notable tenderness. Assessing active abduction against gravity or with a remote examiner helps identify weakness in the abductor muscles [23,42].

Knee Physical Exam With Telemedicine

During a knee examination, the patient's gait should be observed. The examiner can visually examine lower limbs; this is to check for alignment, muscle atrophy, deformities, surgical marks, scars, skin issues, swelling, bruising, or redness. The patient should rotate to allow the camera to view all sides of the knee for a complete inspection. They should then indicate the area of most pain with one finger. Finally, ROM should be tested, noting symmetry and any tenderness across the entire site [35]. A virtual goniometer can help assess alignment and ROM [40,43,44]. The lever test can be adjusted where the examiner applies downward pressure on the distal femur while their other fist supports the calf is especially suitable and applicable for telehealth practice [44-45].

To examine meniscal injury, patients may perform the bounce, hyperflexion, and Thessaly tests; the latter can be done while being in front of the camera. When the patient is seated and facing forward, the J-sign can be observed and evaluated for patellofemoral joint injury. It is possible to self-assess patellofemoral crepitus, particularly when kneeling against resistance, with the help of a family member [35].

Clinical effectiveness

Telehealth has proven to be an important tool in orthopedic practice, as it can offer effective solutions for remote consultations and follow-ups, facilitating outpatient treatment, and providing accessible rehabilitation services. This approach enhances patient convenience and can improve the efficiency of care delivery [10,14,46,47]. 

Buvik et al.'s RCT [10] compared telemedicine services with in-person outpatient visits using specialist questionnaires (primary outcome) and also analyzed complications, referral patterns, surgeries, and consultation times. Of the 400 randomized patients, 199 underwent telemedicine consultations and 190 underwent in-person consultations. The specialist score was slightly better in the in-person group (1.72 vs 1.82, p = 0.003), but within the non-inferiority margin. Surgeons rated 98% of video consultations as “good” or “very good.” They concluded that telemedicine is a safe option for selected orthopedic patients, although further evaluation of cost and patient satisfaction is needed before wider adoption. 

Vuolio et al. [14] studied whether videoconference consultations and traditional outpatient visits differ in executing patient management plans over a one-year follow-up. They randomized 145 orthopedic patients (84 first visits, 61 follow-ups). They found no significant difference in the implementation of treatment plans between groups and concluded that videoconferencing is a valid alternative to in-person specialist consultations. Aarnio et al. [48] found that 92% of surgeons felt their decisions made via teleconsultation were equivalent to those made during in-person outpatient visits.

Ohno et al. [49] report a practical telehealth experience between a Japanese base and Antarctica. From 1956 to 2003, 4,932 remote consultations were done, averaging four per winter crew member. Nearly half addressed surgical and orthopedic issues; others covered some other medical problems. With advances in satellite systems, transmission of images and real-time telemedicine became feasible. They concluded that telehealth is an effective means of providing remote medical support in isolated settings.

Haukipuro et al. [11] explored videoconferencing for orthopedic outpatient examinations in an RCT, assigning 76 patients to video consults at a primary care unit and 69 to in-clinic visits. They found that telemedicine was technically feasible, patient satisfaction was similar in both groups, and those who used video consultations were more willing to repeat them. Despite more examination challenges via video, they conclude that videoconferencing is usable when imaging is not critical.

Wallace et al. [50] observed a significant observation where patients who participated in the virtual outreach group were offered follow-up appointments more frequently than those who were followed up with using classical follow-up. Another study reported that a significantly higher proportion of patients assessed by an emergency medicine specialist using telemedicine were offered a follow-up consultation [51]. 

Baxter et al. [52] highlighted that telemedicine practice is very important to high-risk patients for total joint arthroplasty (TJA). These patients, ineligible for ambulatory surgery, received personalized preoperative counseling through face-to-face, video, or phone appointments. The study found that neither phone nor video consultations were inferior to clinic or face-to-face consultations. Furthermore, patients who received video consultations were more likely to be discharged home and had lower 30-day readmission rates compared to those who had phone consultations. While patient self-selection might have influenced these results, they suggest that the effectiveness of telemedicine may vary depending on the delivery method.

Iyer et al. created a best practices technique for conducting a spine physical examination via telemedicine, including modified methods for assessing strength, sensation, and specific tests like the straight leg raise, Spurling’s test, and tests for myelopathy. Although many spine experts agree that they can provide safe and appropriate remote care for patients with lumbar stenosis, lumbar radiculopathy, and cervical radiculopathy, further research is necessary to validate examinations for conditions such as cervical myelopathy [53,54]. 

**Figure 1:**
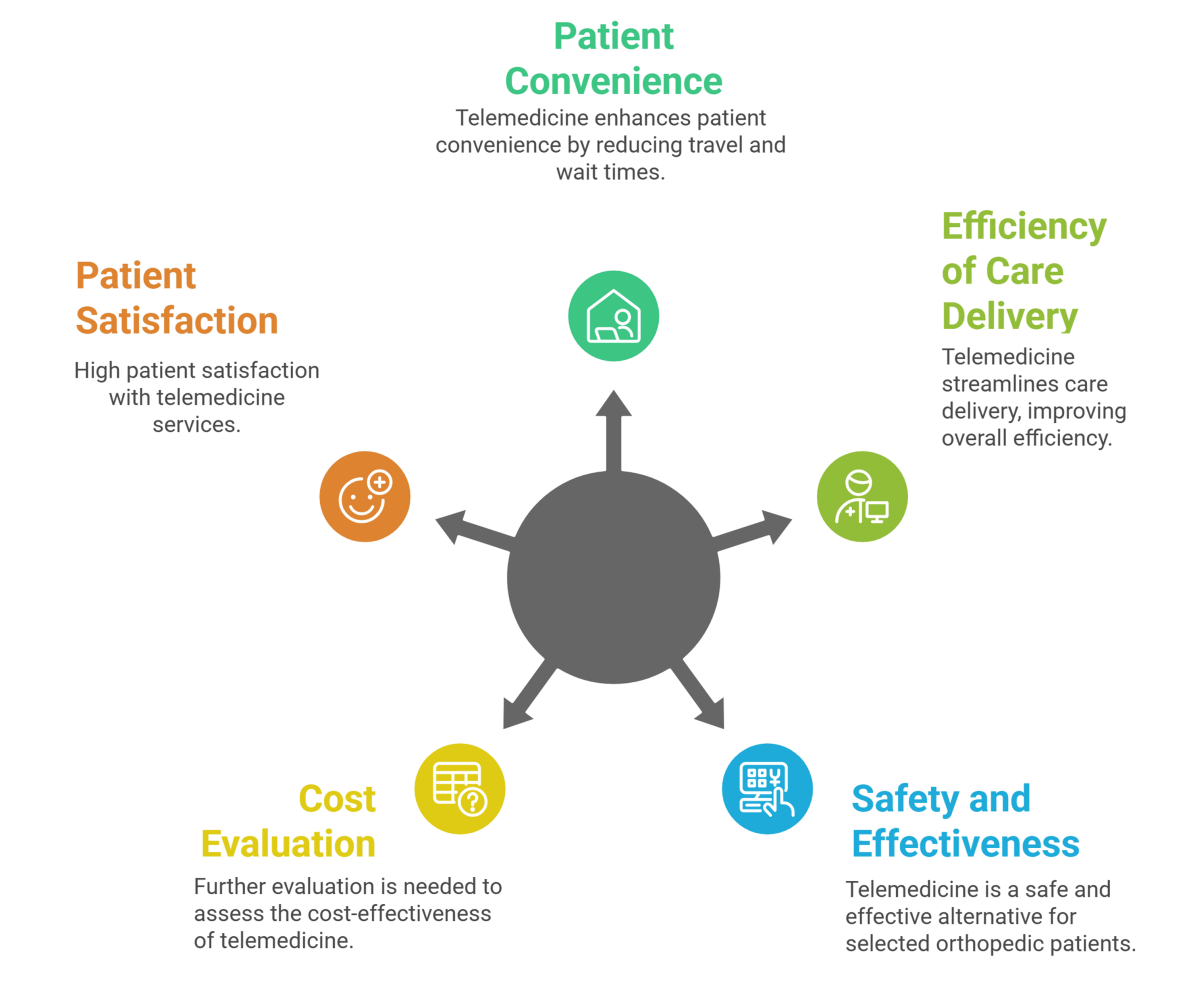
Clinical effectiveness of telemedicine Figure created by article authors summarizing the clinical effectiveness of telemedicine. Source: [50-55].

Farid et al. assessed the use of telemedicine for screening adolescents with idiopathic scoliosis, finding strong agreement between telehealth and outpatient or clinical measurements, including trunk rotation angle. Both patients and caregivers reported high satisfaction and willingness to recommend the telehealth service (Figure 1) [55].

Limitations 

While a hands-on examination is ideal for a thorough musculoskeletal assessment, including a detailed ROM, palpation, and strength testing, certain specialized tests like the Lachman or pivot-shift for ACL tears or load and shift for shoulder instability cannot be replicated remotely. Nevertheless, many aspects of a sports medicine physical exam can be effectively conducted through an online telehealth platform [35]. 

Some patients may experience discomfort with telehealth consultations or remote postoperative follow-up visits, as mentioned by one study that revealed a 12.1% dropout rate among patients assigned to telehealth visits following rotator cuff surgery. Similar rates have been observed in other studies, indicating a notable and important challenge in patient adherence to remote care models [10,56-58]. Many studies have highlighted the critical role of transmitting X-ray images of sufficient quality [13,59]. 

Virtual care presents several challenges, particularly in ensuring fair access for various racial and socioeconomic groups. A study by Xiong et al. compared new orthopedic patient demographics over 10 weeks in 2019 with a similar period during the early pandemic that found unequal access to telemedicine, with Hispanic and Asian patients having fewer telehealth appointments than White patients. Furthermore, patients with Medicaid insurance used virtual care less often than those with private insurance [60-62].

A survey by Puzzitello et al. revealed that patients with lower incomes and poorer health experienced greater challenges using telehealth for orthopedic care during the COVID-19 pandemic. Furthermore, non-White patients and those with government insurance reported a higher incidence of inadequate care or technical issues after telemedicine appointments in pediatric orthopedic and sports medicine settings [63,64].

**Figure 2:**
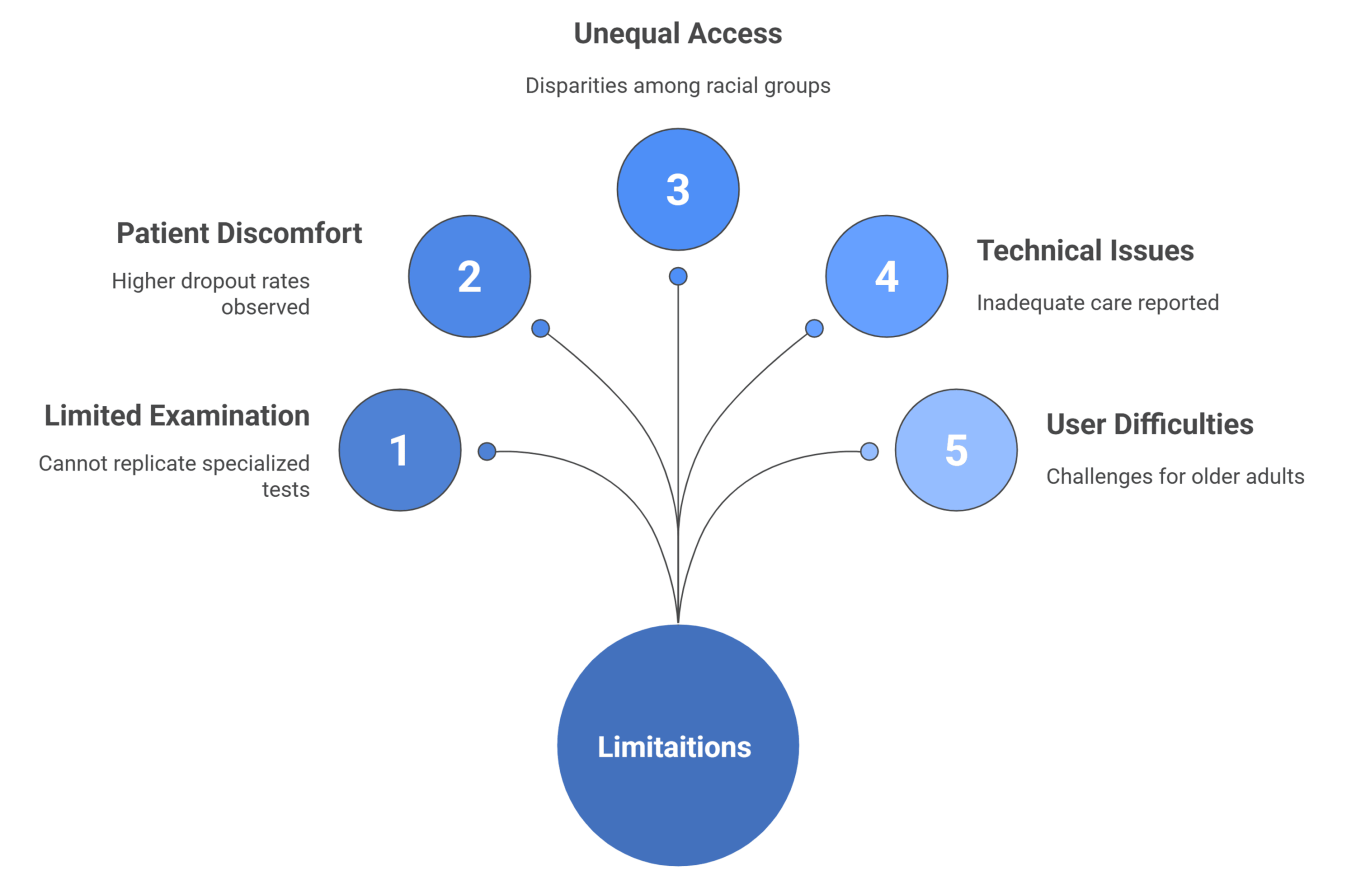
Limitations of telemedicine in orthopedic practice This figure was created by the article authors based on references [56-65].

Many studies highlighted that most groups that need telemedicine, such as older people, patients with chronic disease, or rural areas, may find difficulties in using telemedicine. For example, a mixed-methods needs assessment by Mao et al. revealed that a primary problem for older adults in independent living facilities was their lack of familiarity with telemedicine technology and which will make it very difficult for them to use. To improve remote clinical encounters, it is crucial to streamline the process by developing specific telehealth protocols that can assess telehealth capabilities and preferences by patients, providing clear step-by-step instructions for accessing appointments, and establishing a system for on-demand technical support (Figure 2) [65].

Future Directions

The ongoing improvements of large language models (LLMs) and artificial intelligence (AI) are expected to bring about further changes in healthcare practices. One promising application is the use of LLM-powered chatbots for initial assessment, suggesting possible diagnoses, and outlining treatment strategies for specific musculoskeletal conditions [1,66]. AI can affect the remote delivery of these services by remotely monitoring patients recovering from surgeries. By using Bluetooth-enabled devices and AI algorithms, data such as mobility, range of motion, patient-reported outcomes, opioid consumption, wound appearance, and rehabilitation compliance can be aggregated to assess patient progress in real time. Further development and validation of these AI tools are expected to improve virtual orthopedic practice [67].

## Conclusions

Across orthopedic subspecialties, telemedicine has matured into a reliable adjunct to in-person care. Modified virtual examinations for the elbow, shoulder, hip, and knee, often using patient-performed maneuvers with household items, enable meaningful assessment, while randomized trials show video consultations are non-inferior for key clinical decisions and patient satisfaction. In routine outpatient follow-up and rehabilitation, telehealth improves access and convenience without compromising safety. Taken together, the evidence supports telemedicine as a permanent, high-quality complement to traditional musculoskeletal practice rather than a wholesale replacement.

Nonetheless, limitations persist. Certain hands-on tests cannot be replicated remotely, some patients disengage from virtual follow-up, and diagnostic quality hinges on transmitting adequate imaging. Equitable access remains uneven across racial, linguistic, and insurance groups, highlighting a digital divide. Strengthening standardized protocols, patient preparation, and technical support can mitigate these gaps. Looking ahead, AI and LLMs, coupled with remote monitoring, can enhance triage, assessment, and longitudinal surveillance - positioning telemedicine to deliver more efficient, personalized orthopedic care while maintaining patient safety and quality.
